# From Linear to Complicated to Complex: Comment on "Using Complexity and Network Concepts to Inform Healthcare Knowledge Translation"

**DOI:** 10.15171/ijhpm.2018.02

**Published:** 2018-01-20

**Authors:** Jo Rycroft-Malone

**Affiliations:** School of Healthcare Sciences, Bangor University, Bangor, UK.

**Keywords:** Knowledge Translation, Complexity, Model, Framework, Collaboration

## Abstract

Attention to collaborative approaches to encouraging evidence use in healthcare practice are gaining traction. The inherent complexities in collaborative and networked approaches to knowledge translation (KT) have been embraced by Kitson and colleagues in their complexity network model. In this commentary, the potential of complexity as presented by Kitson et al within their model is considered. The utility of such a model will be contingent upon how easy users find it to understand and apply to their challenge, and doing so in a way that is useful to not only help with explanation, but also with prediction.

Worldwide, the challenge of encouraging evidence informed practice and service delivery persists. Attention on complexity and collaboration in relation to knowledge production, implementation and evaluation within healthcare is escalating.^1-4^ Arguably this is in part a response to the limited impact that traditional perspectives on evidence and its use have had; there is a growing consensus that “evidence alone does not solve problems.”^4^ The shift away from a pipeline, linear view of evidence use, which usually fails to acknowledge the influence of context, and that real-world problem-solving is complicated and dynamic, has resulted in a growing interest in co-productive conceptualisations.^5^ Whilst the idea of co-production is not new, see for example Van den Ven’s^6^ text on ‘Engaged Scholarship,’ within healthcare these type of approaches have only relatively recently gained some traction. As such, whilst the theoretical evidence base is growing, with some exceptions,^4,5,7^ there has been less attention to what is required to operationalise approaches that require collaboration and interaction. As such, Kitson and colleagues’ efforts to embrace and represent the inherent complexities in a collaborative and networked framing of knowledge translation (KT) are timely.^1^

Kitson and colleagues rightly put forward a central argument ‘*that in order to progress the science and practice of KT in healthcare, we need to re-conceptualize the way we think and talk about translation*.’^1^ Whilst there have been many advances in the field, it has been argued that there has been much repetition,^4^ with scholars rehearsing similar arguments but framed from their disciplinary context and using their particular nomenclature. For example, the central tenets of Rogers theory of diffusion of innovations originally merging from rural sociology in the 1960s have permeated the vocabulary and theorising of numerous KT theories and frameworks over many years.^8-10^ Despite recasting concepts and theories, the ‘magic bullet’ for enabling evidence informed healthcare remains stubbornly illusive. Hence, there continues to be an appetite for ‘*lateral thinking’*^1^ about the KT challenge. Complexity and complexity theory offers a new window and fresh way of thinking about the KT challenge, and is gaining some momentum.^1,4,7^

As acknowledged by Kitson et al, the idea that KT is more complex than linear is not new. The challenge to early conceptualisations of evidence-based practice that focussed attention on the capacity and capability of individual practitioners to find appraise and apply evidence is now well rehearsed. The fact that KT is dynamic, multi-faceted and therefore, complicated, is largely accepted, at least by those who spend time practicing and studying in the field. Additionally, some conceptual frameworks represent KT as more complicated than simple.^11^ However, the challenge is in developing a conceptual framework or theoretical model in a way that both represents the complexities of KT, but which also provides some practical support. This will in part be a result of how complexity is both conceptualised and applied. In general, when scholars have stated that KT is complex, their explanation does not tend to extend beyond recognising that there are multiple components, which interact dynamically, and that action is contextually situated. Therefore it is helpful that Kitson and colleagues have explicitly identified the characteristics of complex adaptive systems they considered relevant to their application. The challenge then is in embedding these characteristics, which are dynamic and interacting, in a representation that is static and 2 dimensional. The utility of such a model will be contingent upon how easy users find it to understand and apply to their challenge, and doing so in a way that is useful to not only help with explanation, but also with prediction.

As a new KT model, which combines network and complexity concepts, the ambition is to ‘…*help design and inform KT initiatives prospectively*....’^1^ Herein lies a challenge. If we accept the core concepts of complexity theory are^7^: self-organisation, interaction, emergence, system history and temporality, ie, that in complex systems there is not a single point of control, that change occurs naturally and continuous as people, knowledge, interventions/programmes and systems interact – arguably, a standard representation is potentially flawed because the situation will be ever-evolving. What represents one set of circumstances, may not represent another in a complex system, although it is possible that whilst self-organisation and emergence cannot be controlled, they can be influenced. Further, ‘*planned change in such a system is also difficult as nothing stands still while we intervene*.’^4^ Whilst the KT processes of planned change identified by Kitson and colleagues are well documented in planned action theories, the test is in translating those into non-linear applications, which work with the system in an emergent way. It is easier to gravitate to a staged, linear process when faced with a complicated challenge because of an illusionary comfort of control. The authors state that their complexity network model represents a dynamic between process and system depending on the needs of a given KT goal, our challenge then is to consider the transferability of the dynamic *their* initiatives created, to a range of different KT situations. The potential is in finding some ‘simple rules’ that might provide a transferable best fit framework/application, however the challenge remains that these simple rules will in themselves be context dependent.

Kitson and colleagues’ paper is a useful contribution in helping to answer the critical question about what the key components are of a system where individuals, groups and organisations work collectively together to solve healthcare challenges based on evidence. Findings from evaluations of policy initiatives to establish collaborations for the purpose of mobilisation knowledge highlight a number of issues.^2,13^ Working collaboratively does not happen in a vacuum. There are certain conditions that make it more or less easy for people to work together productively. Whilst conditions set the context for collaborative knowledge generation and use, the ability of individuals, teams and organisations to meaningfully connect may need to be engineered, in that it is unlikely to happen without planned consideration and intervention. Even where there is a structure and governance framework that facilitates the connection of relevant stakeholders, there will remain a number of different boundaries that will need to be bridged or negotiated – including those between organisations/divisions/departments, between different philosophical perspectives people have about knowledge, its provenance and its mobilisation, between people because of different understandings about meaning and language, and between different groups. These findings reveal an intricate fabric of people, relationships, and contexts that influence knowledge production and use. However, it is important that we do not over-complexify the challenge – something that Kitson et al also raised. Arguably our shift away from KT being less linear to being multi-faceted has resulted in a proliferation of frameworks and theories that pay attention to this complexity, but which have not necessarily helped to successfully guide practical action. We are all keen to find some simple practical solutions to complex problems.

Finally, Kitson et al leave us with a number of questions to consider if we are to change the way we think about KT to recognise the inherent complexities of working collectively to solve problems based on evidence. In contributing to the dialogue encouraged by the authors, the following questions add to their list:

- How do we incentivise and reward people within their respective systems to engage in more collaborative approaches to KT? – for example, career structures of researchers tend not to reward localised knowledge generation, they tend to prioritise international excellence, research done at scale, and academic outputs.
- How should we develop and embed training and education pathways to engender the skills and capabilities required to successfully navigate more co-productive ways of working?
- In a funding environment that tends to operate as a research pipeline, how should funders respond to the challenge of more collaborative and co-productive ways of undertaking research?
- How should we develop our evaluation methods and approaches to better meet the challenges of working with complexity so that we can develop an evidence base that will be transferable across contexts and at scale?

## Ethical issues

Not applicable.

## Competing interests

Author declares that she has no competing interests.

## Author’s contribution

JRM is the single author of the paper.
